# Automation in monitoring gases and particulates in the pulp and paper industry

**DOI:** 10.1155/S1463924684000262

**Published:** 1984

**Authors:** T. L. C. de Souza

**Affiliations:** Pulp and Paper Research Institute of Canada (PPRIC) 570 St. John's Boulevard Pointe Claire Quebec H9R 3J9 Canada


Journal of Automatic Chemistry, Volume 6, Number 3 (July–September 1984), pages 142–147

### Automation in monitoring gases and particulates in the pulp and paper industry

**T. L. C. de Souza**

*Pulp and Paper Research Institute of Canada (PPRIC), 570 St. John's Boulevard, Pointe Claire, Quebec, Canada H9R 3J9*

In all major industries increasing importance is placed on the use of instruments that analyse process streams effectively and efficiently for long periods of practically unattended operation. This helps the process operator to keep an inventory of the materials leaving the process, and check the operating efficiency. In the field of pollution, it helps to confirm that the process is operated within the confines of usually stringent government regulations, in the form of set emission limits. The use of continuous or automated instruments usually cuts back on manpower requirements, while rigid maintenance becomes necessary because of the complexity of operating some of these sophisticated pieces of equipment.

The Canadian pulp and paper industry is required to monitor the pollution it generates of water, air and land. This paper deals with what this industry has done, and is trying to do, in automating the monitoring of pollutant gases and particulate matter.

### Brief kraft pulping process

The principal wood-chip pulping process is called kraft (see figure 1). Here, an aqueous solution of sodium sulphide ($Na_2S$) and hydroxide is used to break down wood-chips, at high temperature and pressure, into fibres which are then washed, bleached and made into paper. The spent reagent, referred to as 'black liquor', now containing unused chemicals and dissolved lignin from the wood, is then concentrated and burnt in the chemical-recovery furnace, the lignin providing useful heat. The process steps of pulping, black liquor oxidation (to reduce pollution) and liquor transportation, oxidize the sodium sulphide to thiosulphate and sulphate. The oxidized chemicals are then reduced in the reducing zone of the recovery furnace back to $Na_2S$ to be reutilized in the pulping step. The hot gases leaving the furnace and containing some hydrogen sulphide, sulphur dioxide, carbon dioxide, water vapour, carbon monoxide and oxygen are often contacted with black liquor in a direct-contact evaporator in order to preconcentrate the liquor prior to burning. The hot gases strip the black liquor of its organic mercaptans, sulphides and disulphides which are the major pollutants carried to the atmosphere via the stack. In certain kraft processes, direct contact between the hot gases and black liquor is avoided and the final concentration of the black liquor is achieved in multiple-effect evaporators. The major gaseous pollutant in such cases is sulphur dioxide ($SO_2$) which causes 'acid rain', a source of great concern to environmentalists. The stack gases also include particulate matter consisting principally of sodium carbonate and sodium sulphate, which, if unchecked, can cause considerable damage to the immediate surroundings.

### Monitoring of gases

The major source of pollutant gases and particulates in a kraft pulping process is from the recovery furnace, followed by the lime-kiln burning operations. Other emission sources are the digester, smelt tank, evaporators, black liquor oxidation tank, washer hood vents and waste-water treatment operations. The pulp and paper industry monitors such pollutant gases as hydrogen sulphide ($H_2S$), methyl mercaptan ($CH_3SH$), dimethyl sulphide $(CH_3)_2S$ and dimethyl disulphide $(CH_3)_2S_2$, together referred to as total reduced sulphur (TRS). Other sulphur gases like $SO_2$ and odourless carbonyl sulphide (COS) are also measured, the latter frequently being formed under overloaded furnace conditions. Gases other than those containing sulphur—usually products of combustion—are sometimes analysed in order to obtain an indication of the extent of combustion. These could be carbon monoxide, carbon dioxide, methane, oxygen and oxides of nitrogen ($NO_x$) usually consisting of a mixture of nitric oxide (NO) and nitrogen dioxide ($NO_2$).

Analysing these gases on a continuous basis is made difficult because they are normally at elevated temperatures and contain significant amounts of moisture and particulate matter. Also, the sulphur compounds are even more difficult due to their reactivity and corrosiveness. Some of the the problems encountered can be eliminated by prefiltering the gases, and maintaining the temperature of the gas above its dew-point at all times or condensing out the moisture. Inert materials, like Teflon, should be used to transport particularly the sulphur gases from the flue duct to the analytical instrument in the least possible time, in order to prevent interaction among the compounds, as between $H_2S$ and $SO_2$, in the presence of moisture.

### *Sample-conditioning devices*

Quite frequently, the sample gas, as drawn from the process stream, is unfit to be led directly into the analytical instruments for reasons of fouling up the inside of the systems, or causing serious errors in measurements due to certain interferences. In such cases, the sample has to be preconditioned in order to achieve uninterrupted analysis for a reasonably long period of time.

Sample handling and conditioning systems are usually designed to extract the sample from the flue gas duct and transfer it to the analytical instrument for subsequent analysis, without changing the concentration or character of the constituents to be

© 1982 Academic Press, Inc.

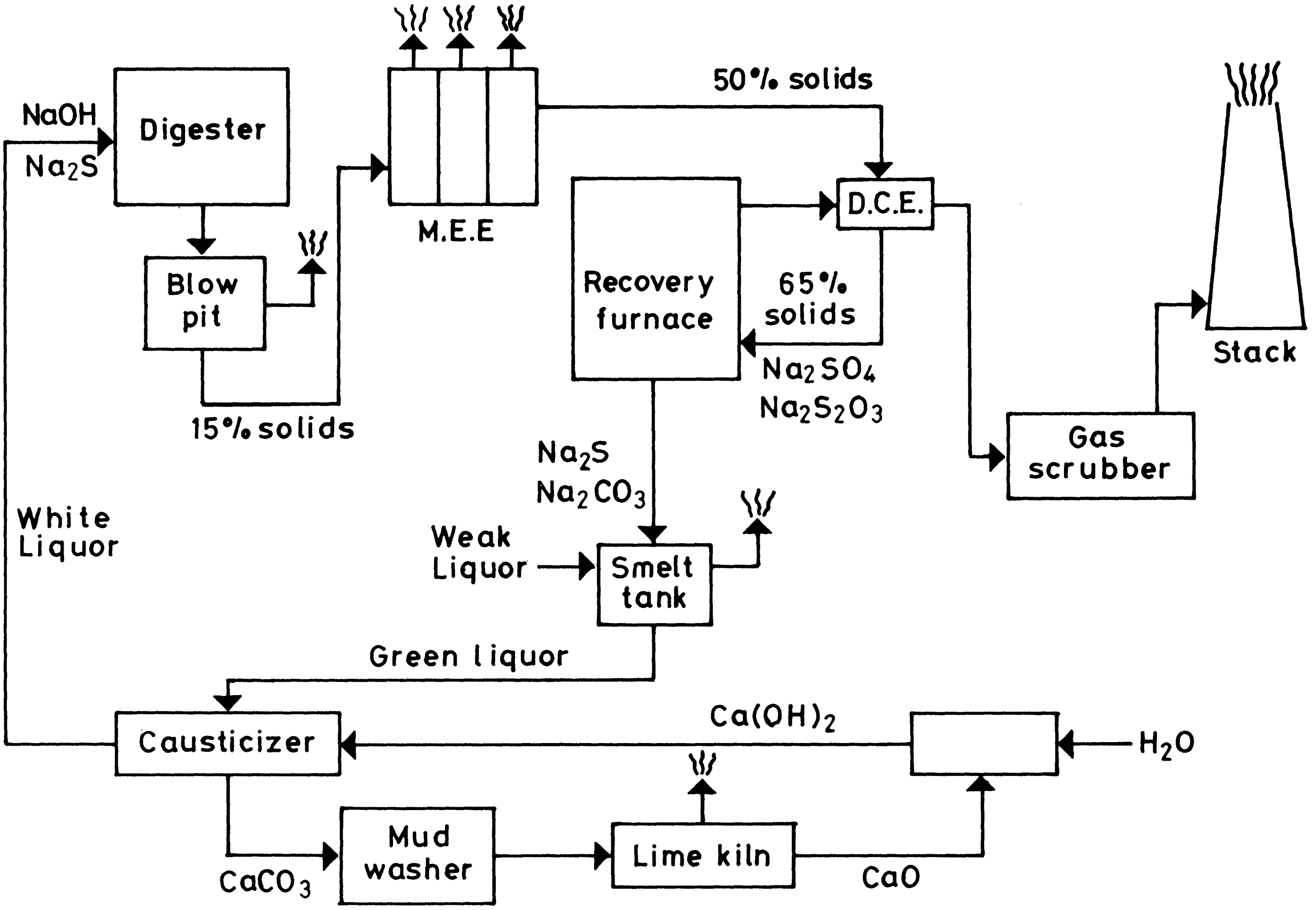

*Figure 1. Schematic diagram of a kraft pulping process.*

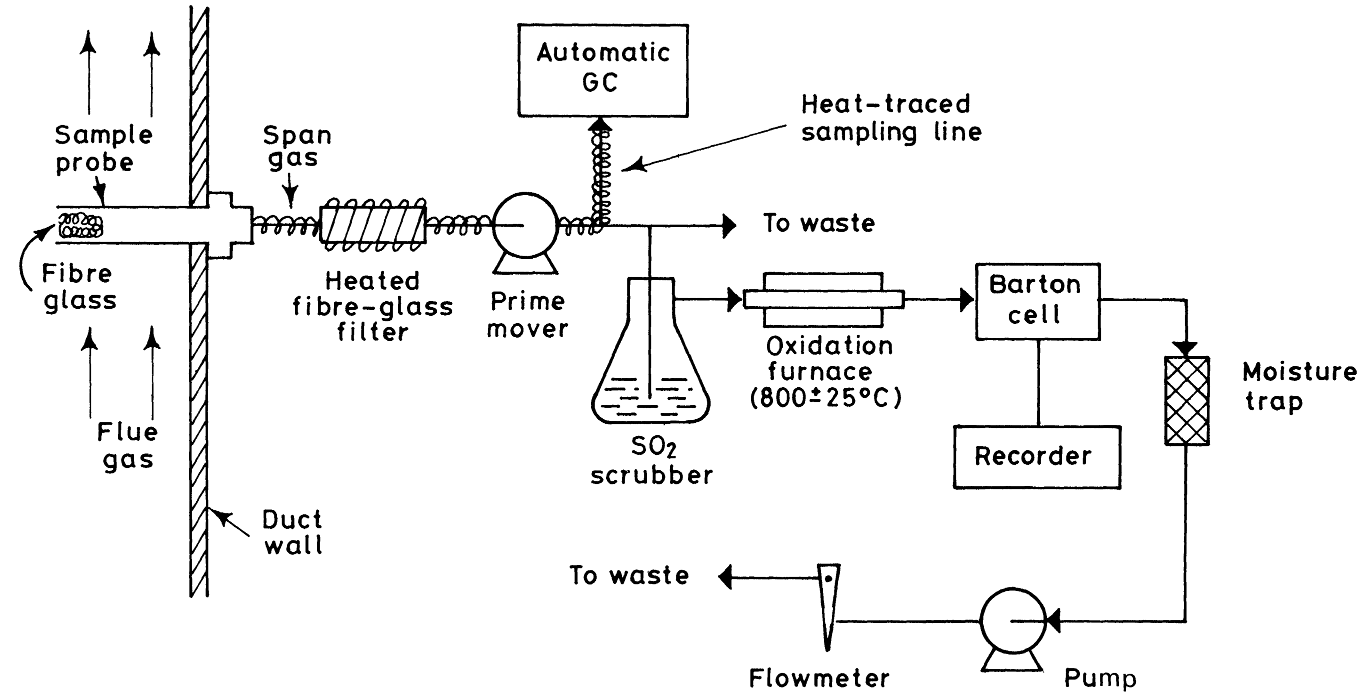

*Figure 2. Typical sampling train used in the measurement of total reduced sulphur (TRS) concentrations.*

measured. Some very useful information about measuring gaseous sulphur compounds can be gathered from a number of publications [1–3], which deal with such topics as materials of construction (Teflon is considered to be the best) and the avoidance of some of the pitfalls. The choice of selecting a sample probe that is fitted with a filter and usually made of stainless-steel or ceramic is covered by Blosser *et al.* [4], and probes equipped with automatic blowback features by Thoen *et al.* [5].

In measuring TRS, it is sometimes necessary to first remove $SO_2$ from the sample gas by the use of a preconditioned liquid scrubbing solution, like potassium acid phthalate, or a mixture of citric acid and potassium citrate, inserted immediately downstream of the sample probe. Depending upon the type of analytical system used, it is also sometimes necessary to convert the TRS compounds to $SO_2$ in an oxidation furnace (figure 2) prior to measurement so that $SO_2$-sensitive detection methods, such as flame photometry, ultra-violet spectrophotometry and electrochemistry, can be used. This conversion helps oxidize potentially interfering organic compounds, such as terpenes and olefinic and aromatic hydrocarbons, into non-reactive carbon dioxide and water. The drawback of this conversion is that non-odoriferous sulphur gases (not to be included in the TRS measurement), like COS, will also be converted to $SO_2$ and erroneously reported as TRS.

Coarse particulates can be removed by either one of the following ways:

(1) Glass-wool placed at the head of the sample probe.
(2) A blowback system employing a sintered stainless-steel or ceramic head placed in a stainless-steel tube.
(3) An 'inertial filter' [6] utilizing the principle of particulates segregating to the middle of a fast-moving gas stream.

Fine particulates can be filtered off by the use of small diameter, pore-size fibre-glass filters, which are placed downstream of the coarse filter.

Water vapour in the sample gas can be separated out by allowing condensation to occur in a catchpot containing an acid such as $H_2SO_4$, which helps to release any sulphur gas, like $H_2S$, that might be absorbed by the condensate, back to the gaseous phase. The alternative is to prevent condensation of the moisture by maintaining the temperature of the gas above the dew-point by either heat-tracing the sample lines, diluting the sample gas with $N_2$ or using a semi-permeable membrane to separate water vapour from other gas constitutents.

### *Continuous sulphur gas detectors*

A number of analytical devices for measuring sulphur gas concentrations in gas streams are available on the market, the most notable of these are based on the principles of flame photometry, ultra-violet spectroscopy and electrochemical sensing.

### *Flame photometers*

Advantage is taken of those compounds like sulphur and phosphorus, which, when burnt in a hydrogen-rich flame, emit characteristic coloured light which is monitored for its intensity and related to the concentration. This principle was first put into practical use by Brody and Chaney [7] in 1966 by developing the flame photometric detector (FPD), which is used with 526 and 394 nm wavelength filters for measuring phosphorus and sulphur respectively. Another version of this single flame detector has been reported by Gangwal [8]: it utilizes two flames and is said to give a more uniform response and to eliminate interference due to high organic background.

The FPD detector can be used with instruments measuring total gas concentrations (non-separating) or individual concentrations (separating). In the former configuration the sample gas is fed continuously to the detector which responds to the particular species it is attuned to. An example of this is the Meloy Sulphur Analyzer [9]. In the second configuration, a mixture of the gases is separated into its individual constitutents before being directed to the detector for measurement. This is usually achieved with gas-separating columns, such as those used in gas chromatography. The columns could be either of the packed or capillary type with high surface-to-volume ratio. The separation of the mixed gases is temperature-dependent and is achieved by exploiting the difference in relative affinities of the individual constituents for the packing material or liquid-phase coating used. The difference in affinities causes the various constituents of the mixture to pass through the column at different rates. An inert carrier gas moves the sample through the column, the weakly absorbed components being the first to emerge. A suitable detector then measures the concentration of each compound as it leaves the column.

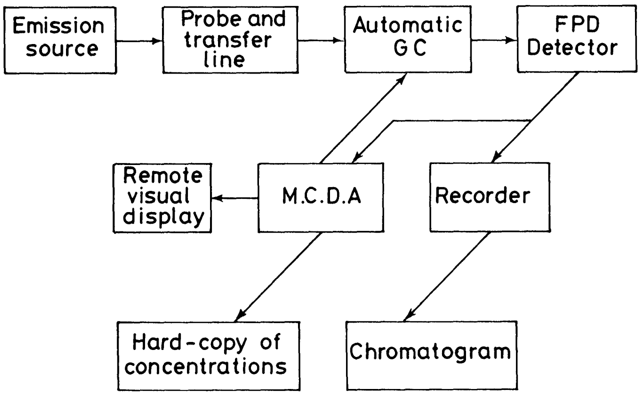

*Figure 3. Schematic diagram of the automatic gas chromatograph developed at the Pulp and Paper Research Institute of Canada for the measurement of total reduced sulphur concentrations.*

Only intermittent analyses can be obtained with gas chromatographs, but the cycle times can be decreased significantly to obtain rapid analyses which are within the reporting time requirements specified by some regulatory agencies. This is usually done with chromatographs that have been automated. An example of this is one dedicated GC for sulphur analysis that was completely developed at PPRIC by de Souza *et al.* [10]. In this analyser (figure 3) the sampling and analytical procedures, data collection, processing and printing and/or display of the results, directly in ppm concentrations, are fully controlled by a microcomputer. A sample of the analysis report of six sulphur compounds is shown in figure 4; the analysis was completed in about 10 min by the use of three separating columns made of specially treated Porapak QS polymer [11] and maintained at isothermal conditions. The sequencing of column changes (figures 5) was achieved with 10- and four-port valves operated by small electric motors. Calibration was performed with permeation tubes and a single-flame FPD detector.

This prototype GC unit was subjected to about 4000 operating hours, 1200 h of which were spent in continuously monitoring actual recovery furnace gases, laden with particulates, moisture and organic compounds. This was done over a period of about three years and the only major difficulties

DAY / HOUR / MIN
26 / 15 / 00

| GAS | START* | MAX* | END* | AREA | PPM |
|---|---|---|---|---|---|
| H2S | 48 | 55 | 66 | 15414 | 9.1 |
| COS | 66 | 74 | 115 | 09299 | 1.2 |
| SO2 | 115 | 133 | 230 | 22549 | 16.8 |
| † RSH | 230 | 270 | 397 | 42926 | 10.6 |
| R2S | 397 | 428 | 481 | 11053 | 2.1 |
| R2S2 | 481 | 536 | 720 | 06423 | 0.7 |
| TRS | | | | | 23.2 |

* Entries are times, in seconds, from time of injection.
† R ≡ $CH_3$

*Figure 4. Teletype print-out of the analysis of gaseous sulphur compounds encountered in the gaseous emissions of a kraft wood pulping process.*

encountered were misalignment of the valves' shafts with those of the electric motors and malfunctions of certain computer 'chips' in the electronic circuits. The separating columns showed no signs of permanent deterioration. This GC is now mass-produced by Western Research and Development of Calgary, Canada. It has been modified to operate with a single temperature-programmed column (specially treated Porapak QS material sold as Supelpak S by Supelco, Inc., of Bellefonte, Pennsylvania, USA) and an updated system of data logging, computing concentrations and printing the results. Other dedicated automatic sulphur GCS on the market are by Tracor Analytical Instruments and Bendix Environmental and Process Instrumentation Division.

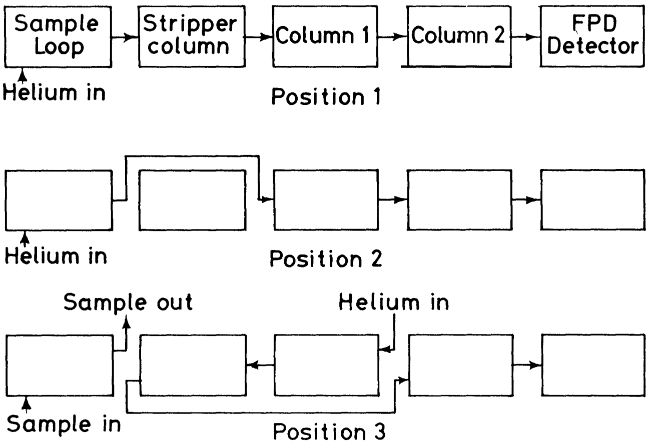

*Figure 5. Schematic diagram of the procedure used with two valves, for the separation and detection of a mixture of gaseous sulphur compounds.*

*Ultra-violet sensors*

These detectors are based upon the principle of ultra-violet (UV) light energy absorption by the sample gas, at a fixed wavelength in reference to another non-absorbing wavelength. Only one gaseous component is measured at any given time. Ultra-violet light transmitted through the sample cell is divided into a direct and reflected beam. In the measuring channel, the beam passes through an optical filter passing only the measuring wavelength, for example 289 nm for $SO_2$, the reference beam being centred around a wavelength of 578 nm. The difference in the two signals obtained from the reference and measuring channels is linear with the gas concentration [12]. As mentioned earlier, organic compounds, like phenols, ethylene, pinene etc., also found in kraft furnace emissions can cause interference. However, they can be eliminated by the use of an oxidation furnace prior to measurement with the accompanying error of including COS, if any, in the TRS measurement.

*Electrochemical analysers*

There are only two well-known instruments in this category that are being used by the Canadian pulp and paper industry; they are listed below:

*Coulometric titrators*: continuous titration devices that provide and measure the amount of reactant needed to satisfy the demand of the reacting sample gas. Sulphur gas analysers of this type measure the electrical current necessary to maintain a fixed low concentration of free halogen, for example bromine, in a halide solution like hydrogen bromide. Figure 6 illustrates the working of the titration cell. Sample gas diffuses into the hydrobromic acid and reacts with available free bromine. The amount of bromine consumed is sensed by a pair of electrodes, which then relay this information to the pair of free bromine-generating electrodes. These provide the necessary amount of electric current needed to make up the difference in free bromine. The current provided is the measure of the concentration of the sample gas.

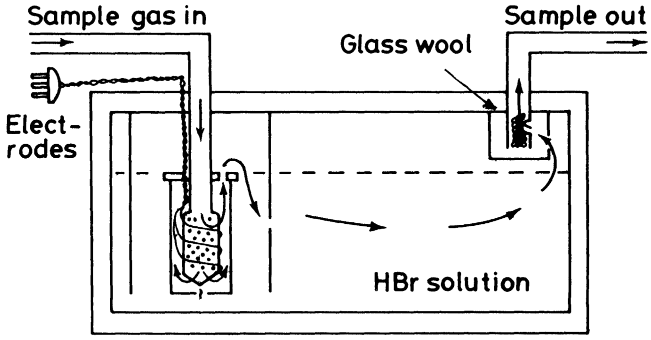

*Figure 6. Cut-away view of a Barton titration cell.*

The instruments are flow-dependent and give unequal responses to equal concentrations of individual sulphur compounds [2]. They are also prone to interference from organic compounds that are emitted from recovery furnaces and which react with bromine. Once again, this interference can be eliminated by sample oxidation, but if odourless compounds like COS are present, then they will be converted to $SO_2$ giving positive responses. As such, COS does not react with bromine.

*Fuel cell sensors*: have been on the market for some time, but have only recently been used by the paper companies to monitor TRS emissions (see figure 7). The detector unit is completely enclosed in a cell containing an electrolytic solution, a semi-permeable membrane and a set of electrodes. Temperature and pressure (positive) in the unit are usually maintained constant. Sample gas is passed over the membrane and the gas of interest, for example $SO_2$, diffuses from the sample stream into the electrolytic solution, creating an imbalance between the sample and reference electrodes. This imbalance is a measure of the amount of $SO_2$ present in the sample gas.

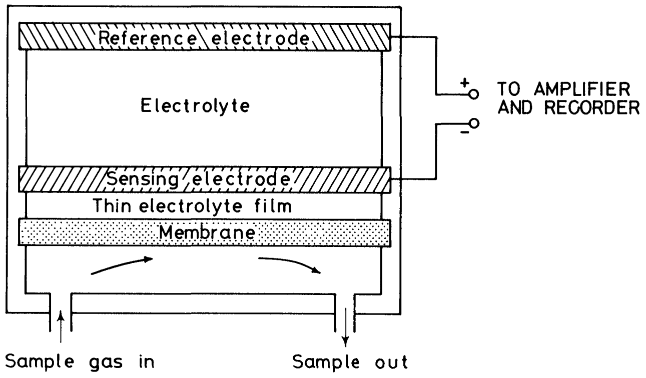

*Figure 7. Cut-away view of a fuel cell used in the analysis of gaseous sulphur compounds.*

The sensor responds to one gas component at a time, so the TRS mixture has to be oxidized to $SO_2$ prior to measurement; with the possibility of accompanying interference from carbonyl sulphide and the like. The detector is said to be relatively interference-free, maintaining stable calibration without substantial drift in response for long periods of time and requiring minimal maintenance [13]. Possible operating difficulties could be due to particulate matter plugging the pores of the membrane, water-vapour condensation in the detector causing erratic response, and sulphuric acid mist, if present, causing severe corrosion. The sensor is temperature- and pressure-dependent and could be subject to delayed response times.

### *Continuous non-sulphur gas detectors*

In this group can be included such gases as $O_2$, $NO_x$, $CO_2$, $N_2$ and CO. Only a few of these are measured on a continuous basis in the industry, although most of them can be monitored without interruption. Only the first two gases will be discussed here.

### *Oxygen*

There are two popular ways of monitoring oxygen from process streams on a continuous basis: by polarography and by exploiting the paramagnetic properties of oxygen.

The sensor of the polarographic instrument contains a silver anode and a gold cathode, both of which are protected from the sample by a thin membrane of Teflon. An electrolyte of KCl solution is also used. Oxygen from the sample gas diffuses through the membrane and is reduced at the cathode when a potential is applied across the electrodes, causing a current to flow, thus:

$$\text{At anode:} \quad 4Ag + 4Cl^- \rightarrow 4AgCl + 4e$$

$$\text{At cathode:} \quad O_2 + 2H_2O + 4e \rightarrow 4OH^-$$

The magnitude of the current generated is proportional to the concentration of $O_2$ in the sample gas.

Instruments are also available that utilize the unique property of $O_2$ to be attracted into a magnetic field (paramagnetic), whereas most other gases are slightly repelled out of a magnetic field (diamagnetic). Thus, by measuring the magnetic susceptibility of a sample gas mixture, its oxygen content can be determined.

### *Nitrogen oxides*

Oxides of nitrogen emissions are not so significant from kraft and sulphite pulp mill sources as from power boilers, because large amounts of water present in the spent cooking liquors and lime mud burnt in the furnaces, inhibit the occurrence of high flame temperatures needed for the formation of significant amounts of nitrogen oxides. However, when measurements are required, the most commonly used continuous techniques to measure nitrogen oxides are electrochemical transducer membrane cells, ultra-violet spectrophotometry and chemiluminescence. With the exception of the latter, the other methods have already been discussed.

In a chemiluminescent $NO_x$ analyser, a preconditioned sample gas is made to react with excess ozone ($O_3$). Any nitrogen oxide present in the sample gas reacts with the ozone to produce light, the intensity of which is proportional to the concentration of NO [14]. Nitrogen dioxide is measured by diverting a parallel sample stream through a catalytic converter where $NO_2$ is reduced to NO, which, together with the original NO present in the sample gas, undergoes the chemiluminescent reaction with $O_3$. The resulting signal, minus that of the original NO, gives the concentration of $NO_2$.

### Monitoring of particulates

Most of the particulates emitted in the pulp and paper industry are from the chemical recovery and lime kiln operations in the pulping process. The loss of these chemical solids not only increases the cost and maintenance of abatement equipment, and the cost of chemicals used, but also pollutes the surrounding atmosphere. Some of the more popular current monitors of particulate matter are based on optical measurements, Beta-radiation attentuation and charge transfer.

### *Optical devices*

These instruments utilize a system whereby a beam of light from a lamp source is directed across a stack gas duct to a detector (single beam) or to a reflector and back to a detector (double beam). The loss in light intensity (converted to an electrical signal) obtained—due to absorption by particulate matter—is then amplified and recorded on a chart recorder. The sensitivity and accuracy of these devices are affected by the intensity and wavelength of light used, path length, moisture content, particle size, colour and particle mass loading of the flue gas stream.

The monitors give indirect mass correlations and have serious limitations. However, they are simple, inexpensive and relatively easy to operate and maintain. They can provide warnings of malfunctions of control equipment and non-conformance with stack opacity standards. Severe vibrations can lead to unstable readings. The lenses need frequent or continuous cleaning because fine dust tends to settle on them. These monitors are best for stacks with low particulate concentrations following high-efficiency control devices and where the particle-size distribution and other physical properties are relatively uniform.

### *Beta-radiation attenuation*

In this technique, a beam of Beta particles (electrons) is passed through a medium such as flue gas, resulting in a net loss in beam intensity due to some absorption and reflection. Such a reduction is called 'Beta-radiation attenuation', and is a measure of the mass of material through which the beam passes. The correlation of Beta attenuation with mass is a function of the ratio of the number of electrons to nuclear mass per molecule. This ratio is between 0·4 to 0·5 for all elements except hydrogen [15 and 16], hence this correlation is virtually independent of the composition of the particle material.

The major problem areas for these devices are in the fields of sampling rates, sample conditioning, sample transfer from the duct to the sensor without significant sample loss and the transport mechanism.

### *Charge transfer*

When an electrically charged particle comes in physical contact with a sensing electrode, the electrical charge is transfered to the electrode. This charge transfer is then measured as a flow of electrical current from the sensor and is the basis of instruments utilizing this principle.

This is a single-point measuring technique with many potential problems, one of which is the disproportionate response to submicron particles which do not contribute proportionally to mass. The sensor is affected by the degree of saturation of the sample gas [17] and is subject to invalid calibrations due to changes in particulate shape and size distribution. Also, the probability and nature of physical contact between the particulates and the sensor depend upon the composition and surface properties of the particulates, the sensor, the flow characteristics of the sample stream and the degree of contamination of the sensing element.

### Data recording and processing

Almost all types of analysers are now equipped with some form of data-gathering system. For continuous monitoring, it is very important to acquire the right type of data reducing and reporting system so as to minimize manual effort and obtain the final print-out in practical units.

Analogue strip or circular-chart recorders are the most commonly used data-gathering devices, the former offer great versatility, the latter being limited by the chart length and poor time resolution.

Digital recorders record intermittently either instantaneous or integrated values, for a given time period. They do not process the data, but can be easily interfaced with a computer for data processing.

Data processors, on the other hand, can not only record data, but also average and compute emission standards, thereby eliminating manual effort of data reducing. Generally, there are two data-processing methods. The first interfaces the analyser with an analogue-to-digital converter which in turn is connected to the mill computer. The latter accepts the digital signals and performs the necessary calculations for the final print-out. The second method is to have a dedicated computer to process only the continuous monitoring data from the analyser; of course, it is more expensive. With these computers, the final report can be set to desired formats often involving provisions to indicate emission rates in process terms and emissions which exceed the amounts or guidelines set by the regulatory bodies for a particular pollutant.

### Acknowledgement

This paper was originally published by Academic Press, Inc. in Dan P. Manka (ed.) *Automated Stream Analysis for Process Control* (1982).

### References

1. Nader, J. S., *Journal of the Air Pollution Control Association*, **23** (1973), 587.
2. de Souza, T. L. C., Lane, D. C. and Bhatia, S. P., *Pulp & Paper Canada*, **76** (1975), 73.
3. National Information Service, US Department of Commerce, *Environmental Pollution Control, Pulp and Paper Industry, Part I Air*, PB-261 708/2SL (1976), 17.
4. Blosser, R. O., Cooper, H. B. H. and Megy, J. A., *Technical Bulletin No. 38* (National Council of the Paper Industry for Air and Stream Improvement, New York, USA, 1968).
5. Theon, G. N., DeHass, G. G. and Austin, R. R., *Technical Association of the Pulp and Paper Industry*, **52** (1969), 2304.
6. Metallurgical Corporation, *The Mott Inertial Filter* (Farmington Industrial Park, Farmington, Connecticut 06032, USA).
7. Brody, S. S. and Chaney, J. E., *Journal of Gas Chromatography*, **4** (1966), 42.
8. Gangwal, S. K., *Journal of Chromatographic Science*, **17** (1979), 196.
9. Meloy Laboratories Inc., Springfield, Virginia 22151, USA.
10. de Souza, T. L. C., Wostradowski, R. A., Poole, R., Vadas, O., Bhatia, S. P. and Prahacs, S., *Pulp & Paper Canada*, **79** (1978), T242.
11. de Souza, T. L. C., Lane, D. C. and Bhatia, S. P., *Analytical Chemistry*, **47** (1975), 543.
12. Saltzman, R. S. and Williamson, J. A., *Monitoring Stationary Source Emissions for Air Pollutants with Photometric Analyzer Systems (E. I. du Pont de Nemours & Co. Inc., Instrum. Div., Wilmington, Delaware 19898, USA)*.
13. Hann, G. K. and Nylund, J. E., *Pulp & Paper Canada*, **80** (1979), T315.
14. Heyman, G. A. and Turner, G. S., Presented at the Instrumentation Society of America Symposium in San Francisco, California, USA (1976).
15. Sem, G. J., Borgos, J. A. and Olin, J. G., *Chemical Engineering Progress*, **67** (1971), 83.
16. Larssen, S., Ensor, D. S. and Pilat, M. J., *Technical Association of the Pulp and Paper Industry*, **55** (1972), 88.
17. Azarniouch, M. K. and Prahacs, S., Evaluation of the IKOR in-stack continuous particulate emission monitor. Presented at The Canadian Pulp and Paper Association's Environmental Improvement Conference held in Moncton, New Brunswick, Canada (1977).

### PITTSBURGH AND COMPUTERS

On 25 February 1985, at the annual Pittsburgh Conference and Exposition, a tutorial workshop is to be presented on PCs in the laboratory, this will be followed by a session on the elements of the new instrumentation science, from microcomputer subsystems in instruments to expert systems for acquisition and interpretation of data. On 26 February the Society for Analytical Chemists of Pittsburgh Award Symposium, 'Chemometrics and process analytical chemistry' honouring Professor Bruce Kowalski, will be held. This will cover the impact of computing in industrial processes and the new opportunities for industrial analytical chemists. Additionally, a panel discussion, held as part of the Monday afternoon symposium, will give the audience the opportunity to discuss developments in their own laboratories.

*More information from David R. Weill III, Shady Side Academy, 423 Fox Chapel Road, Pittsburgh, Pennsylvania 15238, USA.*

